# Nutritional, Oxidative, and Sensory Quality of Whiteleg (*Penaeus vannamei*) and Pink Shrimp (*Penaeus paulensis*) From Different Production Systems During Frozen Storage

**DOI:** 10.1111/1750-3841.71194

**Published:** 2026-06-18

**Authors:** Sarita Correa Rosa, Milena Padilha, Bibiana Alves dos Santos, Pamela Cristiele Oliveira Trindade, Géssica Hollweg, Manoela Meira Balzan, Priscila Rossato Fracari, Alexandre José Cichoski, Roger Wagner, Natalia Fernandes Pereira, Giovanni Lemos de Mello, Márcio Vargas‐Ramella, Paulo Cezar Bastianello Campagnol

**Affiliations:** ^1^ Universidade Federal de Santa Maria Santa Maria Brazil; ^2^ Mar do Brasil Aquicultura Laguna Brazil; ^3^ Universidade do Estado de Santa Catarina (UDESC) Laguna Brazil

**Keywords:** aquaculture systems, fatty acid profile, omega‐3 polyunsaturated fatty acids, oxidative stability, sensory analysis, shrimp quality

## Abstract

This study compared the nutritional composition, fatty acid profile, oxidative stability, and sensory quality of whiteleg shrimp (*Penaeus vannamei*) and pink shrimp (*Penaeus paulensis*) obtained from semi‐intensive, organic, and wild systems. Four groups were evaluated: Semi‐intensive *P. vannamei* from Santa Catarina and Ceará (Brazil) (PV1 and PV2, respectively), organic *P. vannamei* (PV3), and wild‐caught *P. paulensis* (PPA). Raw and cooked samples were analyzed for proximate composition, pH, color, and fatty acids at the start of storage, and for thiobarbituric acid reactive substances (TBARS) and total volatile basic nitrogen (TVB‐N), alongside sensory acceptance and Check‐All‐That‐Apply (CATA) profiling, over 90 days of frozen storage. Organic and wild shrimp exhibited higher omega‐3 polyunsaturated fatty acids, particularly EPA and DHA, and more favorable n‐6/n‐3 ratios than semi‐intensive counterparts, but were also more prone to oxidation during storage. Semi‐intensive shrimp, especially PV2, maintained greater oxidative stability and visual quality over time. CATA test revealed a progressive shift from positive descriptors (e.g., fresh aroma and characteristic flavor) to negative attributes associated with oxidation (e.g., unpleasant aroma and color), particularly in PPA after prolonged storage. However, these changes were not consistently reflected in consumer acceptance, which declined over time but showed no significant differences among treatments for most consumers. Findings demonstrate that production system and region influence shrimp quality, with organic and wild systems offering superior nutritional profiles but lower oxidative stability. These results underscore the need for targeted antioxidant strategies in lipid‐rich systems and support informed choices in shrimp farming to balance health benefits, sensory quality, and storage stability.

**Practical Applications:**

This study can help shrimp producers, processors, and retailers choose production and storage strategies that balance nutritional value, appearance, and eating quality. Organic and wild shrimp may offer more omega‐3 fatty acids, but they may need better protection against quality loss during frozen storage. The results can guide feed formulation, antioxidant use, and storage decisions to deliver shrimp products that remain nutritious and acceptable to consumers.

## 1 Introduction

Aquaculture is the fastest‐growing sector of the farmed food industry, with annual growth rates (Saengsuk et al. 2024). Aquaculture production, including both marine and freshwater species, has now exceeded wild‐catch levels. Among these species, shrimp holds a prominent position in farmed seafood worldwide due to consumer demand for their physicochemical composition and sensorial attributes (Z. Liu et al. 2021).

According to the FAO, world aquaculture production reached 96 million tons (live weight) in 2023 (FAO 2023a), with farmed shrimp accounting for 5.6 million tons. This production is projected to grow by 4.8% in 2024 (FAO 2023b). In this global context, Brazil ranks as the world's 8th largest shrimp producer. In 2023, national farmed shrimp production reached approximately 127,500 tons, a 13% increase from the previous year, generating an estimated value of R$2.63 billion (≈ US$486 million). Production is predominantly concentrated in the northeast region, which is responsible for 99.6% of the country's output, with the state of Ceará alone accounting for 57% of total production (MPA 2025; Seafood Brasil 2024).

In consonance with this, aquaculture is increasingly focusing on innovative systems and technologies that minimize environmental impacts (H. Yang et al. 2025). This approach aligns with global sustainability goals, particularly the United Nations Sustainable Development Goals (SDGs), which emphasize the need to meet rising demand for animal protein (SDG 2: Zero Hunger), combat climate change (SDG 13: Climate Action), and promote the sustainable use of aquatic environments (SDG 14: Life Below Water) (Jiang et al. 2022). Consequently, aquaculture can play a crucial role in contributing to global food security while alleviating the overexploitation of natural resources.

Beyond sustainability, consumers and the food industry are increasingly concerned with the quality, nutritional value, taste, texture, and freshness of aquaculture products (Z. Yang, Jiang, et al. 2024). In this sense, studies evaluating aquatic animal products from different farming systems have focused on their proximate composition and sensory attributes. Among shrimp species, whiteleg shrimp (*Penaeus vannamei*), the most widely farmed species (accounting for about 80% of global production), is favored for its rapid growth, resilience to adverse conditions, and high nutritional value (Panini et al. 2017; Z. Yang, Jiang, et al. 2024). Concerning their nutritional profile, *P. vannamei* proved to be a good source of protein, essential amino acids, and minerals such as calcium, iron, and zinc, as well as vitamin B12. In addition, they contain high levels of n‐3 polyunsaturated fatty acids (PUFAs), including eicosapentaenoic (C20:5n−3, EPA) and docosahexaenoic (C22:6n−3, DHA) acids (Gonçalves et al. 2021; Panini et al. 2017; C. Zhang et al. 2019).

In contrast, pink shrimp (*P. paulensis*) is an important fishery resource with a continuous distribution in the Western Atlantic (12°S–38°S). In Brazil, *P. paulensis* is the most exploited marine shrimp species, with the south–southeast coastline serving as the primary habitat for adult specimens (C. de Carvalho et al. 2023). In 2023, the average catch of pink shrimp in Brazil was recorded at 712 tons (MPA 2023). *P. paulensis* inhabits shallow waters (*e.g*., lagoons and bays) and fishing areas between 40 and 80 m depths. Known for their dietary flexibility, these shrimp are generalist and opportunistic feeders, consuming a diverse range of food, including insects, polychaetes, microcrustaceans, bivalves, gastropods, algae, seagrasses, and mangrove vegetation (C. de Carvalho et al. 2023). Studies have shown that *P. paulensis* is a rich source of fatty acids, particularly palmitic acid (16:0), stearic acid (18:0), oleic acid (18:1 n‐9), arachidonic acid (ARA, C20:4 n‐6), EPA, and DHA, exhibiting a higher proportion of PUFAs compared to saturated fatty acids (SFAs). Furthermore, the fatty acid profile of wild‐typed animals is not only nutritionally valuable but also useful as a chemical biomarker for understanding their ecological interactions and niche occupation in aquatic environments (Gonçalves et al. 2021). This is especially important because the feeding behavior of pink shrimp has been investigated only for commercial cultivation purposes (Carvalho et al. 2021). Understanding the ecological interactions of *P. paulensis* is also crucial for developing an ecosystem‐based approach to predict environmental impacts and guide public policy.

Considering the factors mentioned above, it is important to note that species, culture region, and diet can significantly influence the quality of shrimp meat (Z. Liu et al. 2021; Panini et al. 2017). Even minor alterations in feed composition can significantly affect product quality. For this reason, studies focused on shrimp production and their products are in constant demand in the global food market. In addition, over the last decade, the rising cost of fishmeal, an important ingredient in shrimp diets, has led to a search for more affordable and sustainable protein sources (Panini et al. 2017). In recent decades, consumer interest in organic food products has emerged. Organic food is generally understood to be food produced in accordance with organic farming regulations that prioritize environmental sustainability, biodiversity, and animal welfare. However, while the consumer perception of organic foods has been extensively studied, there is relatively little published data comparing the nutritional value and sensory attributes of organic versus conventional foods (Ladwein and Sánchez Romero 2021).

Despite growing consumer interest in organic aquaculture and the increasing adoption of sustainable farming practices, few studies have directly compared shrimp from different production systems (semi‐intensive, organic, and wild) while simultaneously analyzing nutritional composition and sensory attributes. Furthermore, the pink shrimp, although ecologically and economically important in Brazil, remains underexplored with respect to its physicochemical and sensory characteristics. It is important to note that comparisons in this study reflect integrated differences among production systems, including species, rather than isolated interspecific effects. Therefore, this study provides a novel, integrated comparison of two shrimp species (*P. vannamei* and *P. paulensis*) harvested from three distinct production systems, assessing their proximate composition, fatty acid profiles, sensory acceptance, and oxidative stability during storage. We hypothesize that feeding regime, farming intensity, and species type significantly influence shrimp quality and consumer perception.

## 2 Materials and Methods

### 2.1 Experimental Design and Materials

To assess the effects of species, culture system, and harvest location on the quality parameters of shrimp, samples were divided into four distinct batches:

- PV1: *P. vannamei* from a conventional semi‐intensive system in Laguna, Santa Catarina, Brazil (28°22′24.6″ S, 48°47′30.5″ W).
- PV2: *P. vannamei* from a conventional semi‐intensive system in Ceará, Brazil (2°52′07.3″ S, 40°00′10.2″ W). The geographical origin of the shrimp was indicated using approximate coordinates, which represent the broader production region rather than the exact collection site.
- PV3: *P. vannamei* from an organic‐certified extensive system in Laguna, Santa Catarina, Brazil (28°31′29.3″ S, 48°50′17.6″ W).
- PPA: *Penaeus paulensis*, wild‐caught from a southern coast lagoon of Brazil (28°24′12.2″ S 48°50′23.7″ W).

Except for the PPA group, PV1, PV2, and PV3 shrimp were farmed in traditional commercial ponds supplied with estuarine (brackish) water with variable salinity, similar to the lagoon where PPA was harvested. The PPA shrimp were wild‐caught from a lagoon with a surface area of 183 km^2^ that flows directly into the Atlantic Ocean. The PV1 and PV2 shrimp were fed with supplemental shrimp feed in addition to natural food sources available in the ponds. In contrast, the PV3 shrimp were reared solely on natural food in an organic‐certified farm, in accordance with Brazilian regulations (MAPA/MPA 2011). The PV1, PV2, and PV3 shrimp were farmed during a production cycle of approximately 100–150 days between October and July.

**FIGURE 1:**
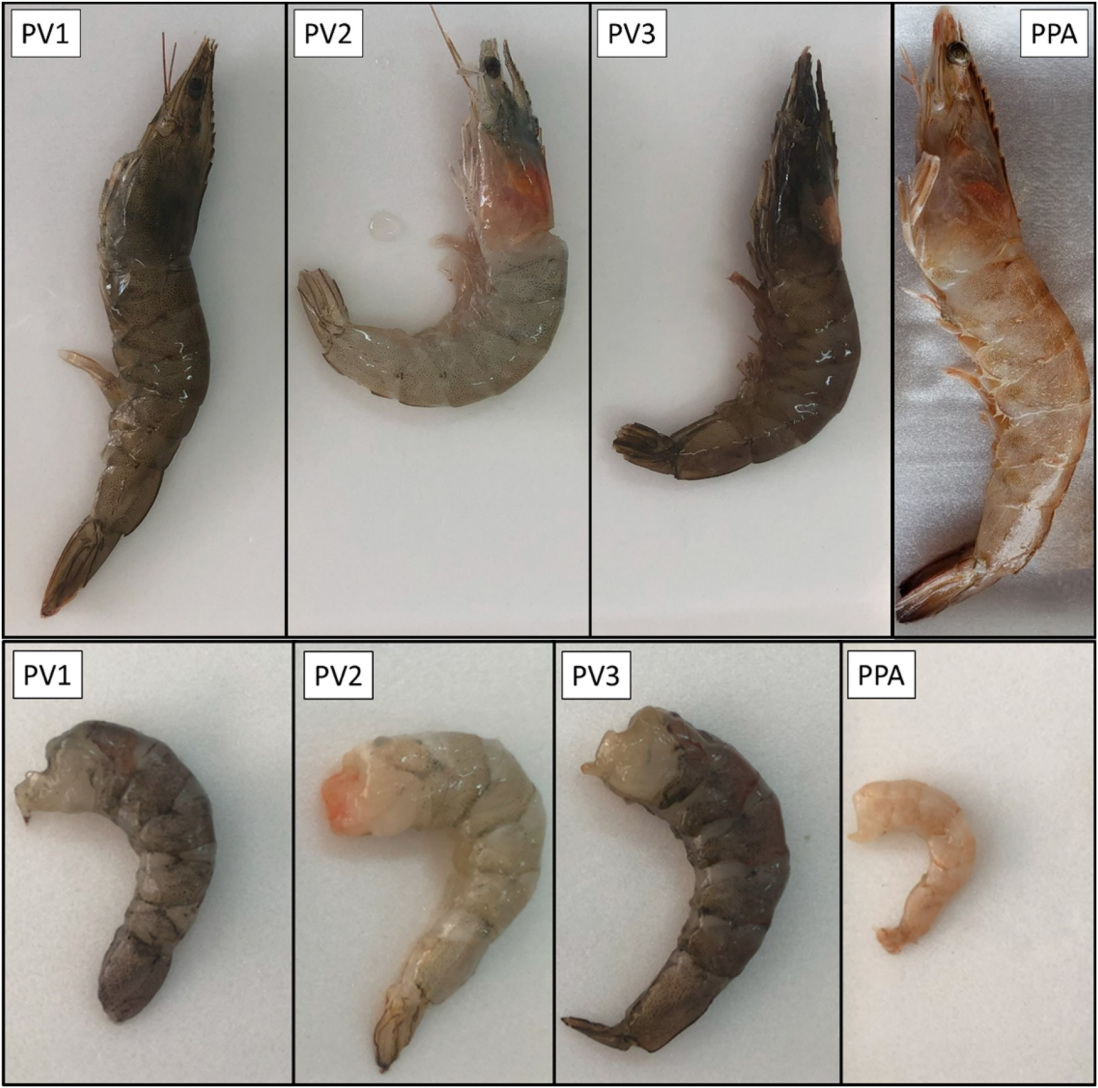
Visual aspect of whole (a) and deveined (b) shrimp: PV1 (*Penaeus vannamei*, semi‐intensive farming, Santa Catarina), PV2 (*P. vannamei*, semi‐intensive farming, Ceará), PV3 (*P. vannamei*, organic‐certified farming, Santa Catarina), and PPA (*Penaeus paulensis*, wild‐caught, Santa Catarina).

The PPA shrimp were harvested (not farmed) between January and June by an independent professional fisherman, in accordance with Brazilian legislation. For each production system, three independent collections were conducted to account for potential variability across the harvest period. In total, approximately 10 kg of shrimp were obtained per treatment. The average individual weight ranged from 15 to 20 g for PV1–PV3 and around 8 g for PPA. The visual aspects of the shrimp samples are presented in Figure 1. After harvesting, samples from each batch were frozen at −20°C and stored for 90 days. The samples were then evaluated for physicochemical characteristics, sensory attributes, and oxidative stability. All analyses, except sensory evaluation, were conducted on both raw and cooked shrimp. For cooking, shrimp were manually peeled and deveined, then immersed in boiling water for 50 s.

### 2.2 Physicochemical Analysis

#### 2.2.1 Proximate Composition, pH, and Instrumental Color (L*, a*, and b*)

At the onset of frozen storage, shrimp samples were analyzed for proximate composition (moisture, ash, protein, and lipid content), pH, and instrumental color (L*, a*, and b*). Proximate composition and pH were determined in triplicate. Lipid content was quantified using the chloroform–methanol extraction method described by Bligh and Dyer (1959). Moisture, ash, and protein contents were assessed according to AOAC (2010) official methods 950.46, 920.153, and 992.15, respectively.

For pH measurement, 5 g of shrimp muscle tissue was homogenized in 50 mL of distilled water. The homogenate was analyzed using a digital pH meter (Model 130 MA; Mettler Toledo, Barueri, SP, Brazil), previously calibrated with pH 4.0 and 7.0 buffer solutions (Merck, Darmstadt, Germany).

Instrumental color parameters were recorded 15 min after sample preparation. Color measurements were conducted using a CR‐400 colorimeter (Konica Minolta Sensing Inc., Osaka, Japan), operating in spectral reflectance mode with a 10° standard observer, D65 illuminant, and a 1.5 cm circular aperture. For each sample, six independent readings were collected.

#### 2.2.2 Fatty Acids Profile

Fatty acid profiles were determined at the beginning of the storage period (Day 1) in both raw and cooked shrimp samples, and all analyses were performed in triplicate. The lipids were extracted according to Bligh and Dyer (1959) with modifications. Briefly, 5 g of previously sampled and ground shrimp were weighed into a 50 mL Falcon tube and added to the extractor solvent mixture: Chloroform (8 mL), methanol (16 mL), and water (2.5 mL, adjusted for sample moisture), and shaken for 1 h. Lipid partitioning was performed after the addition of chloroform (8 mL) and sodium sulfate aqueous solution (1.5%), followed by centrifugation at 4676 × *g* for 5 min. Subsequently, approximately 20 mg of lipids from the chloroform extract were esterified according to the American Oil Chemists' Society (AOCS 1997) with adaptations. Derivatization to methyl esters was performed by adding 0.2 mL of KOH (2 M) methanolic solution and 2 mL of hexane, and the mixture was incubated in a water bath at 40°C for 5 min. Then, 3 mL of saturated sodium chloride solution was added, and the mixture was centrifuged at 3575 × *g* for 5 min. Fatty acid methyl esters (FAMEs) were analyzed using a gas chromatograph equipped with a flame ionization detector (GC‐FID; Varian 3400 CX, CA, USA). One microliter of the FAME extract was injected in split mode (1:20) at 250°C. Hydrogen (99.999% purity) was used as the carrier gas at a constant pressure of 20 psi. Separation was achieved using a ZB‐FFAP capillary column (60 m × 0.25 mm i.d.; 0.25 µm film thickness). The oven temperature was programmed as follows: 50°C for 0.5 min, then increased to 180°C at 10°C/min, to 220°C at 2°C/min, and finally to 235°C at 10°C/min, where it was held for 10 min. The FID temperature was maintained at 250°C. Fatty acids were identified by comparing experimental retention times with commercial standards (FAME Mix 37 [P/N 47885‐U], vaccenic trans acid [P/N 46905‐U], and docosapentaenoic acid [P/N 47563‐U], Sigma‐Aldrich, USA). Results were expressed as a percentage of the total chromatographic area, considering FID response factors and ester–acid conversion factors (Visentainer 2012).

### 2.3 Thiobarbituric Acid Reactive Substances and Total Volatile Basic Nitrogen

Thiobarbituric acid reactive substances (TBARS) and total volatile basic nitrogen (TVB‐N) were analyzed at 1, 30, 60, and 90 days of frozen storage. All determinations were conducted in triplicate. TBARS was evaluated using the method described by Bruna et al. (2001), with results expressed as milligrams of malondialdehyde per kilogram of sample (mg MDA/kg). TVB‐N content was quantified following the method proposed by EC (2005). Results were expressed as milligrams of nitrogen per 100 grams of muscle (mg N/100 g).

### 2.4 Sensory Properties

Sensorial analysis was conducted on Days 1, 45, and 90 of frozen storage, with 100 consumers participating on each evaluation day. All participants received full information regarding the objectives, procedures, and potential risks of the study and provided written informed consent before participation. The study protocol was approved by the Ethics Committee of the Federal University of Santa Maria (UFSM), under CAAE number 92604325.0.0000.5346. Participation was entirely voluntary, without coercion, and participants retained the right to withdraw at any time. The rights, privacy, and confidentiality of all individuals were safeguarded, and no vulnerable populations were included.

The evaluation included a hedonic acceptance test followed by a Check‐All‐That‐Apply (CATA) task. In the acceptance test, participants rated the attribute of overall liking using a nine‐point unstructured hedonic scale, ranging from 1 (“*disliked extremely*”) to 9 (“*liked extremely*”). Immediately after, consumers completed the CATA task by selecting all descriptors that they perceived as representative of each sample.

The CATA attributes were generated through a consensus‐based approach involving eight researchers with experience in sensory analysis, who evaluated the samples and defined descriptors based on the sensory characteristics perceived, ensuring that the final list included relevant, representative, and non‐redundant terms, following established practices in consumer‐based sensory studies (Jaeger et al. 2020). The list of CATA attributes included terms related to color (pinkish/orange color, pale color, bright color, dull color, unpleasant and pleasant color), aroma (fresh aroma, ammonia‐like aroma, sweet aroma, characteristic aroma, mild aroma, pleasant aroma, and unpleasant aroma), flavor (sweet flavor, salty flavor, bitter flavor, mild flavor, marine flavor, earthy flavor, unpleasant flavor, metallic flavor, characteristic flavor, and pleasant flavor), and texture (firm texture, soft texture, fibrous texture, rubbery texture, and juicy texture).

Shrimp samples were cooked in boiling water for 50 s, as previously described, then individually wrapped in aluminum foil and kept warm before serving. Samples were presented monadically in a randomized order, using three‐digit‐coded plastic cups to minimize bias (Macfie et al. 1989). All sensory sessions were conducted in individual booths under controlled conditions, including fluorescent lighting (∼350 lux) and ambient temperature. Water and unsalted crackers were provided for palate cleansing between samples.

### 2.5 Statistical Analysis

Physicochemical parameters were analyzed using mixed‐effects linear models. Each treatment comprised three independent biological replicates, corresponding to independent collections conducted throughout the harvest period. Proximate composition, pH, fatty‐acid percentages, TBARS, and TVB‐N were determined in analytical triplicate per biological replicate; instrumental color (L*, a*, b*) was based on six readings per sample, yielding 18 measurements per treatment × cooking condition. Treatment was included as a fixed effect; where applicable, storage time, cooking condition, and their interactions with treatment were also included as fixed effects. For TBARS and TVB‐N, storage time was treated as a within‐subject factor, with biological replicate (nested within treatment) included as a random intercept to account for repeated measurements and between‐collection variability. Fatty‐acid data were modeled separately for raw and cooked samples. Residual normality (Shapiro–Wilk test) and homogeneity of variances (Levene's test) were verified prior to inference. When significant fixed effects were detected, means were compared using Tukey's HSD test with Satterthwaite approximation of denominator degrees of freedom. Statistical significance was set at *p* < 0.05 (95% confidence level).

For sensory data, the frequency data obtained from the CATA test were analyzed using Cochran's *Q* test to identify significant differences in attribute selection among treatments within each storage time, followed by multiple pairwise comparisons using the Marascuilo procedure. Correspondence analysis (CA) was then applied to visualize patterns in term selection across treatments. Sensory acceptance data were analyzed using a mixed‐effects linear model, with treatments as fixed effects and individual consumers as random effects. In addition, a cluster analysis was performed to identify distinct consumer segments based on individual overall liking scores, and subsequent analyses were conducted within each cluster to account for consumer heterogeneity. Tukey's test was used for post hoc comparisons with a 5% threshold for statistical significance. All statistical procedures were carried out using XLSTAT software version 2019.1 (Addinsoft, Paris, France).

## 3 Results and Discussion

### 3.1 Proximate Composition, pH, and Instrumental Color

Table 1 presents the proximate composition (moisture, protein, lipid, and ash), pH, and instrumental color (L*, a*, b*) of raw and cooked shrimp from four different treatments: PV1 (semi‐intensive farmed *P. vannamei*—SC), PV2 (semi‐intensive farmed *P. vannamei*—CE), PV3 (organic *P. vannamei*—SC), and PPA (wild *P. paulensis*—SC). In the raw samples, moisture content varied significantly among treatments, with PPA showing the highest value and PV3 the lowest. The elevated moisture content in PPA may be related to its wild origin, characterized by slower growth, smaller muscle fibers, and higher water‐binding capacity, which are commonly observed in non‐farmed aquatic animals (H. Yang, Yuan, et al. 2024; Y. Zhang, Xu, et al. 2025). In contrast, the lower moisture in PV3 could be associated with the absence of formulated feed and the reliance on natural productivity in the organic system, which may result in reduced muscle hydration due to limited dietary intake (H. Zhao et al. 2018). The intermediate values observed in PV1 and PV2 suggest that semi‐intensive farming with commercial diets promotes a balanced profile in terms of tissue development and water content.

**TABLE 1:** Chemical composition (moisture, protein, lipid, and ash), pH, and instrumental color (L*, a*, b*) of raw and cooked shrimp.

		PV1	PV2	PV3	PPA	SEM	Sig
Moisture	Raw	76.0^abA^	79.2^abA^	74.5^bA^	80.9^aA^	0.71	*
	Cooked	77.9^bA^	78.7^bA^	75.8^cA^	79.8^aB^	0.35	***
	SEM	0.19	0.05	0.2	0.06		
	Sig	n.s.	n.s.	n.s.	*		
Protein	Raw	21.3^aA^	18.5^bA^	21.8^aA^	15.0^cB^	0.75	***
	Cooked	21.9^aA^	19.2^bA^	22.1^aA^	19.1^bA^	0.33	**
	SEM	0.13	0.08	0.07	0.21		
	Sig	n.s.	n.s.	n.s.	**		
Lipid	Raw	1.7^aA^	0.7^bB^	0.5^bB^	0.9^bA^	0.1	**
	Cooked	2.0^aA^	1.1^bA^	0.9^bA^	1.2^bA^	0.1	***
	SEM	0.02	0.03	0.03	0.01		
	Sig	n.s.	*	*	n.s.		
Ash	Raw	1.3^aB^	1.2^abB^	1.4^aB^	0.8^bB^	0.06	**
	Cooked	1.7^aA^	1.3^bcA^	1.5^abA^	1.0^cA^	0.06	***
	SEM	0.03	0.01	0.01	0.01		
	Sig	**	*	*	*		
pH	Raw	6.85^cB^	7.12^aB^	7.09^bB^	7.13^aB^	0.03	***
	Cooked	6.92^dA^	7.25^aA^	7.15^cA^	7.20^bA^	0.03	***
	SEM	***	***	***	***		
	Sig	0.03	0.06	0.03	0.04		
L*	Raw	42.4^cB^	50.4^bB^	38.7^dB^	54.4^aB^	1.5	***
	Cooked	63.74^cA^	74.7^aA^	62.2^cA^	68.^bA^	0.3	***
	SEM	***	***	***	***		
	Sig	0.79	0.93	0.84	0.54		
a*	Raw	3.7^aB^	1.0^cB^	1.6^cB^	2.7^bB^	0.2	***
	Cooked	19.2^aA^	9.1^dA^	15.5^bA^	13.^cA^	0.2	***
	SEM	0.6	0.3	0.3	0.4		
	Sig	***	***	***	***		
b*	Raw	1.3^bB^	1.7^bB^	0.6^bB^	3.8^aB^	0.3	***
	Cooked	12.5^abA^	9.7^bA^	13.0^abA^	14.6^aA^	0.1	**
	SEM	0.4	0.3	0.4	0.3		
	Sig	***	***	***	***		

*Note*: Shrimp were obtained from four production systems—semi‐intensive farming of *Penaeus vannamei* in Santa Catarina (PV1) and Ceará (PV2), organic farming of *P. vannamei* in Santa Catarina (PV3), and wild‐caught *P. paulensis* in Santa Catarina (PPA), all from Brazil. Different lowercase letters in the same row and uppercase letters in the same column indicate significant differences among treatments (raw or cooked) and between cooking conditions, respectively, according to Tukey's test (*p* < 0.05).

Protein content also showed distinct differences among raw samples. PV3 and PV1 had the highest values, followed by PV2, while PPA exhibited the lowest. The elevated protein content in PV3 may be explained by the slower, but more stable growth conditions in organic systems, which favor protein accumulation in muscle tissue (Beg et al. 2024). PV1, fed with formulated diets under controlled conditions, also demonstrated high protein levels, likely reflecting enhanced nutrient conversion and muscle development. In contrast, the reduced protein content in PPA can be attributed to the natural feeding regime and the energetic demands of wild environments, which often lead to lower protein deposition and greater individual variability (Bhatti et al. 2025). PV1 showed significantly higher protein content than PV2 (*p* < 0.05), despite both being semi‐intensively farmed *P. vannamei*. This highlights the impact of regional conditions, such as feed composition, water temperature, and metabolic rates, on muscle development.

Regarding lipid content, PV1 had the highest concentration among the raw samples, while PV2, PV3, and PPA had significantly lower, but statistically similar, values. The higher lipid levels in PV1 reflect the contribution of formulated feeds, which are typically enriched in energy‐dense ingredients (Zidan et al. 2024). In contrast, PV2 may have used feed with a different composition or feeding regimes, resulting in reduced fat accumulation, as seen in shrimp fed fish‑free diets, which exhibited significantly lower lipid concentration compared to those on conventional feeds (Ray et al. 2019). The difference in lipid content between PV1 and PV2 further reinforces how location‐specific feed practices or nutrient availability can affect fat deposition, even within the same production model. The low lipid content in PV3 is expected due to the absence of supplementation. Meanwhile, the intermediate value in PPA may result from an omnivorous diet based on natural prey with variable lipid profiles, as supported by studies noticing substantial differences in total lipid content between wild and farmed shrimp (Paramaraj et al. 2015).

Ash content in the raw shrimp followed a similar trend. PV3 and PV1 showed the highest values, while PPA had the lowest. The higher mineral content in farmed shrimp is likely influenced by the composition of both the diet and the pond water, which can be enriched with trace elements and macro‐minerals (Simon and Maulianawati 2025). In contrast, the mineral profile of wild shrimp tends to be more variable and dependent on environmental conditions (Raymond et al. 2020), which may explain the lower ash values observed in PPA.

In particular, the proximate composition differences observed between PV1 and PV2 may also reflect intrinsically mediated differences driven by regional climatic variations. The PV2 shrimp cultivated in Ceará (Northeast Region, Brazil), a tropical climate characterized by consistently warmer water, likely experience higher metabolic rates, which can promote lower lipid and protein accumulation. In contrast, PV1 farming in the cooler waters of Santa Catarina (South Region, Brazil) may impose metabolic constraints that slow growth and favor higher lipid and protein retention. This interpretation is supported by studies (Barajas‐Sandoval et al. 2024) showing that thermal environment exerts a strong influence on shrimp metabolism and nutrient partitioning. Thus, regional climatic context, beyond diet and culture system, is likely a significant factor influencing the proximate composition differences between PV1 and PV2.

Furthermore, as assessed in the present work, previous studies evaluating the proximate composition of farmed *P. vannamei* typically reported protein levels in the 21.1%‐22.3% range, with total lipid levels varying depending on feed (Li et al. 2021). Similarly, prior research on pink shrimp (*Penaeus brasiliensis* and *P. paulensis*) in Brazil also indicated low total lipid content with high relative PUFA content in this species (Sánchez‐Camargo et al. 2011). Our data (Table 2) align with these trends and underscore that the organic/wild groups concentrate omega‐3s but may exhibit lower absolute lipid or distinct water content profiles. While direct comparisons are limited due to the unique design of our study (comparing species, systems, and regions), our specific findings for each parameter are consistent with and supported by the broader literature on shrimp quality.

**TABLE 2:** Fatty acid profile (% of total fatty acids) of raw and cooked shrimp.

	Raw							Cooked					
	PV1	PV2	PV3	PPA	Sig	SEM		PV1	PV2	PV3	PPA	Sig	SEM
C14:0	0.73^bc^	0.37^c^	1.4^ab^	1.6^a^	**	0.17		1.6^a^	0.46^c^	1.6^a^	0.72^b^	***	0.16
C15:0	0.44^b^	1.9^a^	1.3^a^	1.7^a^	**	0.18		1.5^ab^	1.7^a^	1.3^b^	0.39^c^	***	0.15
C16:0	20.9^a^	23.4^a^	20.1^a^	21.4^a^	n.s.	0.65		21.1^a^	22.2^a^	20.7^a^	21.5^a^	n.s.	0.35
C16:1	2.3^c^	0.98^c^	4.2^b^	5.8^a^	***	0.56		5.6^a^	0.93^d^	4.5^b^	2.1^c^	***	0.56
C17:0	1.5^c^	2.2^b^	3.4^a^	2.7^b^	***	0.22		2.6^b^	2.1^c^	3.1^a^	1.4^d^	***	0.19
C18:0	10.2^b^	11.2^b^	13.1^a^	12.6^a^	***	0.37		12.7^a^	8.7^a^	12.0^a^	10.8^a^	n.s.	0.73
C18:1 n9	13.7^a^	16.4^a^	9.2^b^	7.9^b^	***	1.1		8.8^b^	18.5^a^	11.0^b^	14.1^ab^	*	1.5
C18:1 n7	3.0^a^	2.6^a^	4.6^a^	3.3^a^	n.s.	0.32		4.4^b^	2.5^d^	5.6^a^	3.2^c^	***	0.36
C18:2 n6	10.4^b^	14.5^a^	5.0^c^	3.1^c^	***	1.4		8.1^c^	15.0^a^	7.4^c^	9.6^b^	***	1.3
C18:3 n6	0.21^ab^	0.08^b^	0.39^a^	0.30^ab^	n.s.	0.04		0.40^a^	0.07^b^	0.39^a^	0.23^ab^	***	0.04
C18:3 n3	1.4^a^	0.72^c^	0.86^bc^	0.98^b^	***	0.07		0.94^c^	0.80^d^	1.3^b^	1.5^a^	***	0.09
C20:0	0.16^a^	0.17^a^	0.32^a^	0.33^a^	*	0.03		0.40^a^	0.29^a^	0.40^a^	0.33^a^	n.s.	0.04
C20:1	0.59^a^	0.47^b^	0.41^b^	0.32^c^	***	0.03		0.29^c^	0.50^b^	0.34^c^	0.65^a^	***	0.04
C20:2	0.99^b^	1.5^a^	0.92^b^	1.1^b^	***	0.08		1.0^b^	1.4^a^	0.74^c^	1.2^b^	***	0.08
C20:3 n6	0.27^a^	0.07^a^	0.06^a^	0.22^a^	*	0.03		0.28^b^	0.20^bc^	0.19^c^	0.37^a^	***	0.02
C20:3 n3	7.3^ab^	4.7^c^	8.2^a^	6.6^b^	***	0.40		6.3^c^	4.6^d^	8.5^a^	7.3^b^	***	0.45
C20:4	0.22^a^	0.07^a^	nd	0.11^a^	n.s.	0.03		0.27^a^	0.06^b^	0.06^b^	0.26^a^	**	0.04
ni	0.62^a^	0.58^a^	0.47^a^	0.61^a^	*	0.02		0.45^b^	0.54^ab^	0.09^c^	0.64^a^	***	0.06
C20:5 n3	11.0^b^	9.2^b^	13.8^a^	14.4^a^	***	0.67		14.2^a^	8.8^b^	12.6^a^	10.4^b^	***	0.64
C22:0	0.10^a^	0.05^a^	0.05^a^	0.11^a^	n.s.	0.02		0.23^a^	0.11^ab^	0.17^ab^	0.06^b^	*	0.02
C22:2	0.79^b^	1.3^a^	1.2^ab^	1.4^a^	**	0.08		1.2^a^	1.2^a^	1.1^a^	3.0^a^	n.s.	0.35
ni	0.22^ab^	nd	0.18^bc^	0.38^a^	**	0.04		0.39^a^	nd	nd	0.15^b^	***	0.05
ni	0.14^b^	nd	0.20^b^	0.39^a^	***	0.04		0.38^a^	0.10^bc^	nd	0.20^b^	***	0.05
ni	0.26^ab^	0.10^b^	0.36^a^	0.21^ab^	*	0.03		0.14^b^	0.15^b^	nd	0.23^a^	***	0.03
ni	0.58^b^	0.09^c^	1.2^a^	0.59^b^	***	0.12		0.45^a^	0.14^b^	nd	0.47^a^	***	0.06
ni	5.4^a^	0.41^a^	0.33^a^	0.84^a^	n.s.	1.1		0.85^a^	0.60^b^	nd	0.96^a^	***	0.11
C22:5 n3	1.9^a^	0.53^b^	1.7^a^	1.8^a^	*	0.20		1.6^a^	0.77^c^	1.7^a^	1.3^b^	***	0.11
C22:6 n3	4.9^b^	6.4^ab^	7.1^ab^	9.4^a^	*	0.63		7.8^a^	5.6^b^	5.2^b^	5.0^b^	***	0.35
∑SFA	36.3^b^	40.3^ab^	43.9^ab^	46.2^a^	*	1.4		45.8^a^	36.4^b^	43.8^ab^	37.3^b^	*	1.4
∑MUFA	19.6^a^	20.4^a^	18.4^a^	17.2^a^	n.s.	0.50		19.1^a^	24.4^a^	21.4^a^	20.0^a^	n.s.	0.99
∑PUFA	39.3^a^	39.1^a^	39.1^a^	39.3^a^	n.s.	0.73		38.1^a^	38.6^a^	39.2^a^	42.3^a^	n.s.	0.64
PUFA/SFA	1.1^a^	0.97^a^	0.89^a^	0.87^a^	n.s.	0.04		0.83^b^	1.1^ab^	0.89^ab^	1.1^a^	*	0.05
∑n‐3	26.4^bc^	21.6^c^	31.6^ab^	33.2^a^	**	1.5		30.8^a^	20.5^c^	29.3^ab^	25.6^b^	***	1.3
∑n‐6	10.9^b^	14.7^a^	5.4^c^	3.6^c^	***	1.3		4.8^d^	15.3^a^	8.0^c^	12.2^b^	***	1.2
n‐6/n‐3	0.41^b^	0.68^a^	0.17^c^	0.11^c^	***	0.07		0.16^c^	0.75^a^	0.27^c^	0.48^b^	***	0.07
AI	0.79^b^	0.64^c^	1.0^a^	1.1^a^	***	0.06		1.1^a^	0.58^c^	0.91^b^	0.70^c^	***	0.06
TI	0.36^ab^	0.48^a^	0.34^b^	0.33^b^	*	0.02		0.35^a^	0.45^a^	0.36^a^	0.39^a^	n.s.	0.02

*Note*: Shrimp were obtained from four production systems—semi‐intensive farming of *Penaeus vannamei* in Santa Catarina (PV1) and Ceará (PV2), organic farming of *P. vannamei* in Santa Catarina (PV3), and wild‐caught *P. paulensis* in Santa Catarina (PPA), Brazil. Different lowercase letters within each row (comparing raw or cooked treatments separately) indicate significant differences (*p* < 0.05) according to Tukey's test. “ni” are presumed to be polyunsaturated isomers of C20 and C22, based on retention time and comparison with literature data, without structural confirmation due to the absence of analytical standards.

Abbreviations: AI, atherogenic index; MUFA, monounsaturated fatty acids; nd, not detected fatty acids; ni, not identified fatty acids; PUFA, polyunsaturated fatty acids; SFA, saturated fatty acids; TI, thrombogenic index.

After cooking, no significant change in moisture was observed in PV1, PV2, and PV3, while PPA showed a reduction. Protein content increased significantly only in PPA. Lipid content rose in PV2 and PV3, and ash content increased in all groups. These changes may be attributed to structural modifications induced by heat, which affect the extractability or concentration of components, depending on the muscle composition and the thermal stability of each group (Verma et al. 2024). In PPA, the increase in protein content after cooking can be attributed to a concentration effect resulting from higher cooking losses in smaller specimens (≈8 g), which also led to proportional increases in lipid and ash contents. This pattern is consistent with water loss during thermal processing, while muscle proteins remain largely retained within the matrix, resulting in higher relative concentrations in the cooked product (Erdogdu et al. 2004; Niamnuy et al. 2008). Despite these effects, the relative ranking among treatments was largely maintained after cooking.

Overall, the results demonstrate that species, rearing system, and feeding strategy play a central role in determining the chemical composition of shrimp muscle in the raw state. Farmed shrimp, particularly those reared in semi‐intensive systems with formulated diets or under organic conditions, exhibited higher levels of protein, lipid, and ash compared to wild‐caught shrimp. Moreover, differences between PV1 and PV2, despite their similar farming systems, underscore the influence of regional conditions, such as climate, salinity, and local feed formulation, on shrimp composition and muscle quality. These differences reflect the influence of nutritional inputs and environmental conditions inherent to each production method. Although cooking induced compositional changes, particularly in lipid and ash content, the magnitude and direction of these effects varied across treatments. Importantly, the relative differences among groups remained consistent after cooking, indicating that the intrinsic characteristics established during cultivation or capture were preserved during thermal processing.

In addition to the differences observed in proximate composition, the pH of raw shrimp varied significantly among treatments (Table 1). PPA and PV2 exhibited the highest values, followed by PV3, while PV1 presented the lowest. The elevated pH observed in PPA may be attributed to postmortem metabolic dynamics commonly seen in wild‐caught crustaceans. Extended time between capture and processing can delay the onset of glycolysis, limit lactic acid formation, and ultimately lead to higher final pH values, similar to patterns reported in wild fish and crustaceans with reduced pre‐mortem glycogen reserves or delayed rigor mortis onset (Cheng et al. 2023; Einen et al. 1998).

In the case of PV2 and PV3, system‐specific factors such as water salinity, feeding regime, and lower metabolic rates typical of extensive or organic farming may have influenced the accumulation and utilization of glycogen pre‐slaughter, thus modulating postmortem acidification and contributing to more alkaline pH levels (Chauhan and England 2018; Dutra et al. 2024). Conversely, the lower pH observed in PV1 could be a result of faster post‐harvest glycolytic activity and earlier onset of rigor mortis, added to the above‐discussed feasible higher glycogen stores in PV1, potentially driven by intensive farming conditions and handling practices that stimulate anaerobic metabolism and subsequent lactic acid accumulation (Chéret et al. 2005). These pH variations between PV1 and PV2 illustrate how regional rearing conditions, even within identical production systems, can influence muscle acid–base balance.

Following cooking, a significant increase in pH was observed in all treatments. This trend is consistent with findings in crustaceans and fish, where thermal processing induces protein denaturation and exposes basic amino groups, thereby reducing proton concentration and elevating pH values (Xiong et al. 2025). Despite the absolute increase, the relative ranking among treatments remained largely consistent, suggesting that intrinsic physiological and environmental differences related to species and production systems were preserved even after thermal processing.

Instrumental color parameters varied significantly among raw samples (Table 1). For lightness (L*), PPA and PV2 exhibited the highest values, followed by PV1, while PV3 displayed the lowest. These findings suggest that both species and rearing systems modulate shrimp muscle optical properties. The darker appearance of PV3 may stem from reduced pigmentation owing to the absence of formulated feed, which limits dietary carotenoid intake. In contrast, the higher lightness in PPA and PV2 likely reflects differences in muscle structure combined with increased carotenoid availability, pigments that shrimp cannot synthesize and must obtain from their diet or environment. This aligns with studies showing enhanced shrimp pigmentation through dietary carotenoid supplementation, such as algal or natural sources, significantly modifying flesh color in cultured shrimp (Phaffia‐derived astaxanthin: lower L*, higher a*) (B. Zhang, Zhang et al. 2025), and seaweed supplementation improving astaxanthin concentration in muscle tissue (Subhra Bikash 2015). The fact that PV2 presented significantly higher L* values than PV1, despite both being semi‐intensively reared *P. vannamei*, suggests that regional factors such as water turbidity, salinity, and feed composition may influence lightness in shrimp muscle.

For redness (a*), PV1 showed the highest value among the raw samples, followed by PPA, while PV2 and PV3 presented the lowest values. The more intense red coloration observed in PV1 may be associated with carotenoid supplementation in the feed, a common practice in semi‐intensive shrimp farming to enhance visual appeal and marketability. Studies have demonstrated that dietary astaxanthin significantly enhances shrimp pigmentation, with higher redness (a*) values observed in shrimp fed astaxanthin‐supplemented diets compared to carotenoid‐free diets (Murniasih et al. 2023; Wade et al. 2017). PPA exhibited intermediate redness, which may be explained by its omnivorous diet in the wild, including algae and microcrustaceans rich in natural pigments. This aligns with research showing that dietary carotenoids strongly correlate with carotenoid accumulation in shrimp tissues, particularly in the exoskeleton, hepatopancreas, and, to a lesser extent, muscle (P. Liu et al. 2025). The low redness values in PV2 and PV3 may therefore indicate lower pigment intake or differences in pigment deposition efficiency. The contrasting a* values between PV1 and PV2, both fed commercial diets, may also reflect differences in the specific type and concentration of carotenoids used by producers in southern versus northeastern Brazil.

In terms of yellowness (b*), PPA displayed the highest value, significantly surpassing the other groups, which were statistically similar. The intense yellow‐orange hue in PPA is likely due to the accumulation of naturally derived carotenoids obtained through its diverse wild diet. This aligns with broader findings that diet, rather than controlled feed, is a primary driver of pigmentation in aquatic animals: Carotenoids synthesized by algae or phytoplankton are transferred through trophic levels, contributing significantly to the coloration of shrimp and other crustaceans (C. C. C. R. de Carvalho and Caramujo 2017). The result reinforces the influence of feeding ecology on pigmentation patterns, even in the absence of formulated dietary supplementation.

After cooking, all color parameters increased significantly across treatments. Lightness, redness, and yellowness values were markedly higher in the cooked state, reflecting pigment release, protein denaturation, and structural changes in the muscle matrix. Despite these shifts, the relative differences among treatments were maintained, with PV2 and PPA exhibiting the highest lightness, PV1 the most intense red, and PPA the highest yellowness. The red/orange color and firm texture have been associated with freshness and good product quality in crustaceans (Panini et al. 2017), and dietary strategies and species‐specific physiology may enhance these attributes. Overall, the results confirm that both nutritional inputs and biological origin are key factors in defining the visual quality of shrimp.

### 3.2 Fatty Acids Profile

Table 2 presents the fatty acid composition of raw and cooked shrimp samples. The fatty acid profile of the raw shrimp revealed a composition highly favorable to human health, marked by elevated levels of PUFA, particularly omega‐3 fatty acids, and low atherogenic and thrombogenic indices. These characteristics are widely recognized for their protective effects against cardiovascular, neurodegenerative, and inflammatory diseases (Banaszak et al. 2024).

Among the SFAs, palmitic acid (C16:0) was the major component in all treatments, with no significant differences among groups. Stearic acid (C18:0) was found at higher levels in PV3 and PPA when compared to PV1 and PV2. Myristic acid (C14:0) varied considerably, being significantly higher in PPA and PV3. The total SFA content was highest in PPA, followed by PV3, PV2, and PV1, showing that wild shrimp tend to have higher levels of saturated fats, though still within nutritionally acceptable limits.

Among monounsaturated fatty acids (MUFAs), oleic acid (C18:1 n‐9) was predominant, with significantly higher concentrations in PV1 and PV2, which were reared under semi‐intensive systems with formulated feed. This pattern suggests that commercial diets likely incorporate vegetable oils rich in oleic acid (Sun et al. 2020). Despite the variation in C18:1 n‐9, the total MUFA content did not differ significantly among treatments, reinforcing the relative stability of this lipid fraction in shrimp muscle tissue.

PUFA accounted for the largest proportion of the total fatty acids in all treatments, with values above 39% and no significant differences among groups. However, relevant differences were observed in key individual PUFAs. Linoleic acid (C18:2 n‐6) was significantly higher in PV2, followed by PV1, and much lower in PV3 and PPA. This is consistent with the use of vegetable oil–based feeds in semi‐intensive systems, which are primary sources of omega‐6 fatty acids (Glencross et al. 2025).

In contrast, omega‐3 fatty acids, especially EPA (C20:5 n‐3) and DHA (C22:6 n‐3), were more abundant in PV3 and PPA. These systems, based on natural productivity or wild prey availability, favor the deposition of long‐chain n‐3 fatty acids in muscle tissue, as evidenced by comparisons between wild and farmed shrimp, where wild specimens consistently show higher EPA and DHA levels (Ouraji et al. 2011). In addition, alpha‐linolenic acid (ALA, C18:3 n‐3), a metabolic precursor of long‐chain n‐3 fatty acids, was significantly more abundant in PV1, likely reflecting its inclusion in commercial feeds. However, crustaceans have limited ability to convert ALA into EPA and DHA effectively, so direct dietary inclusion of EPA/DHA is more critical (Glencross et al. 2025).

The total n‐3 fatty acids were significantly higher in PPA and PV3, intermediate in PV1, and lowest in PV2. Conversely, n‐6 fatty acids followed the opposite pattern, with the highest values in PV2 and PV1 and significantly lower levels in PV3 and PPA. Consequently, the n‐6/n‐3 ratio was significantly lower in shrimp from organic and wild systems, a desirable feature from a nutritional standpoint, as low values are associated with anti‐inflammatory effects and prevention of chronic diseases (Gutierrez‐Guerra et al. 2025).

Nutritional indices such as PUFA/SFA, n‐6/n‐3 ratio, atherogenic index (AI), and thrombogenic index (TI) confirmed the high nutritional quality of the shrimp. Although PUFA/SFA did not differ significantly among treatments, all values were close to or above 1, indicating a balanced lipid profile (Chen and Liu 2020). AI values ranged from 0.64 (PV2) to 1.10 (PPA), while TI values varied between 0.33 (PPA) and 0.48 (PV2). Although statistically different (*p* < 0.05), all samples exhibited AI and TI close to or below the recommended limit of 1.0, as proposed by Ulbricht and Southgate (1991), indicating favorable implications for cardiovascular health. These findings reinforce that shrimp from all production systems possess nutritionally beneficial lipid profiles, consistent with the characteristics of functional foods.

After cooking, the pattern of variation among treatments was preserved, with only minor changes in individual fatty acids. Levels of EPA and DHA remained elevated, and nutritional indices remained favorable, with PUFA/SFA and n‐6/n‐3 ratios compatible with cardioprotective dietary profiles. These results demonstrate that the lipid profile of shrimp is largely resistant to degradation by boiling, maintaining its nutritional value even after culinary processing.

In summary, the data confirm that shrimp, regardless of production system, are an excellent source of health‐promoting fatty acids, especially long‐chain n‐3 PUFAs. Organic (PV3) and wild (PPA) shrimp showed superior profiles in terms of EPA, DHA, and n‐6/n‐3 ratio. At the same time, semi‐intensive systems contributed higher levels of MUFA and n‐6 fatty acids, reflecting the influence of formulated feeds. Interestingly, when comparing PV1 and PV2, both reared under similar semi‐intensive systems, but in different geographic regions, differences emerged. PV1 showed higher levels of ALA and n‐3 precursors (C20:3 n‐3, C22:5 n‐3), and a lower n‐6/n‐3 ratio, suggesting a more balanced, potentially anti‐inflammatory lipid profile. In contrast, PV2 had significantly higher linoleic acid (C18:2 n‐6), total n‐6 PUFA, and a lower AI, likely reflecting a diet richer in vegetable oils or region‐specific feed formulations.

The fatty‐acid patterns in our study, notably higher EPA and DHA and lower n‐6/n‐3 ratios in PV3 (organic) and PPA (wild), and elevated C18:1 n9 and linoleic acid in some semi‐intensive groups, agreed with the literature showing clear dietary imprinting of shrimp muscle lipids. Previous multi‐species and regional studies in Brazil (Gonçalves et al. 2021) reported similar magnitudes of EPA/DHA in wild pink shrimp and consistently higher n‐3 proportions in wild‐caught penaeids compared with typical commercial feed‐based farmed shrimp. These differences underscore how regional variables, including feed composition, ingredient sourcing, and water chemistry, can significantly influence shrimp lipid quality, even within the same farming model. Our findings, therefore, confirm the expected mechanistic link between feed/source and fatty acid profile and provide novel paired evidence across *P. vannamei* and *P. paulensis* within the same regional context.

### 3.3 TBARS and TVB‐N Levels During Frozen Storage

**FIGURE 2:**
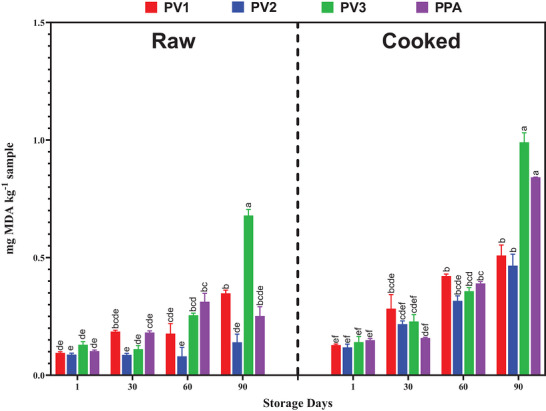
Thiobarbituric acid reactive substances (TBARS) values in raw and cooked shrimp from different treatments during frozen storage. Treatments × Storage time (*p* < 0.05). Different letters indicate significant differences according to Tukey's test. Treatments: See Table 1.

**FIGURE 3:**
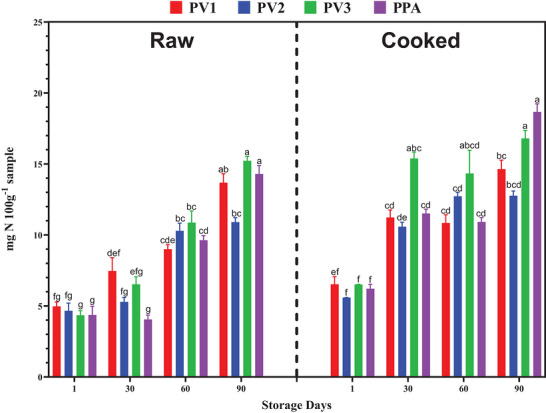
Volatile basic nitrogen (TVB‐N) values in raw and cooked shrimp from different treatments during frozen storage. Treatments × Storage time (*p* < 0.05). Different letters indicate significant differences according to Tukey's test. Treatments: See Table 1.

The progression of TBARS and TVB‐N levels, in both raw and cooked shrimp during frozen storage for up to 90 days, is presented in Figures 2 and 3, respectively. A significant interaction between treatment and storage time was observed (*p* < 0.05), indicating that lipid oxidation dynamics differed across the production systems and storage duration.

In raw shrimp, TBARS values were initially low across all treatments (≤ 0.13 mg MDA/kg at day 1), reflecting good initial lipid stability. However, a marked increase was observed over time, especially in samples from the organic system (PV3), which reached 0.67 mg MDA/kg after 90 days, the highest among all raw samples. Similar patterns were seen in PV1 and PPA, though with slightly lower final values, whereas PV2 exhibited the slowest oxidation rate, maintaining the lowest TBARS concentrations throughout storage. In cooked shrimp, TBARS values were also low at Day 1 (0.11–0.14 mg MDA/kg), but increased more markedly by day 90 in PV3 (0.99 mg MDA/kg) and PPA (0.81 mg MDA/kg), suggesting that frozen storage followed by cooking decreased the oxidative stability.

These oxidation results are consistent with the previously discussed fatty acid profile (Table 2). Treatments PV3 (organic) and PPA (wild), which exhibited the highest susceptibility to lipid oxidation during frozen storage, also showed significantly higher levels of long‐chain omega‐3 PUFAs, particularly EPA and DHA. These fatty acids are highly prone to peroxidation due to their multiple double bonds (Zaloga 2021). In contrast, samples from PV2 and PV1, which had lower omega‐3 content and higher levels of MUFA and n‐6 PUFA, showed reduced oxidative degradation. Among these, PV2 demonstrated slightly greater oxidative resistance than PV1 in raw shrimp at Day 90, suggesting that regional variables, such as feed formulation or environmental conditions, may further influence oxidative stability, even within similar farming systems.

TVB‐N values followed a similar trend. In raw shrimp, the TVB‐N concentrations increased progressively during storage. The highest TVB‐N levels at Day 90 were observed in PV3 (15.2 mg N/100 g) and PPA (14.3 mg N/100 g), indicating greater protein degradation and volatile amine formation. In cooked shrimp, TVB‐N values were generally higher than in their raw counterparts, especially after 30 days, with PPA reaching the highest value (18.7 mg N/100 g) at the end of storage. The lowest TVB‐N contents at Day 90 were found in PV1 and PV2, paralleling their lower TBARS values and reflecting reduced oxidative and proteolytic activity. Differences in shrimp size among treatments, which reflect inherent characteristics of species and production systems, may also have contributed, to some extent, to variability in deterioration rates observed during storage.

### 3.4 Sensory Properties

Sensory analysis was conducted using CATA and an acceptance test over frozen storage periods (1, 45, and 90 days). No visual evidence of melanosis (e.g., localized black spots on the exoskeleton) was observed during sample preparation or analysis.

**FIGURE 4:**
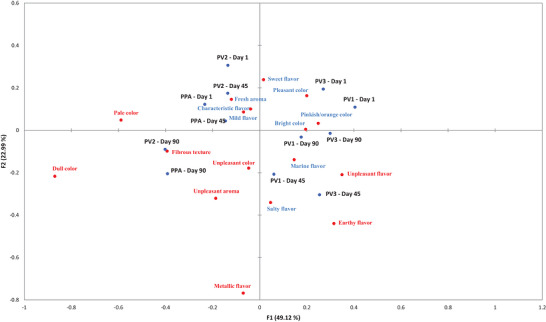
Sensory profiles of shrimp samples during frozen storage based on correspondence analysis of CATA descriptors. Treatments: See Table 1.

The CA of CATA descriptors for shrimp samples over storage (Figure 4) revealed changes in sensory attributes during frozen storage. At Day 1, all treatments (PV1, PV2, PV3, and PPA) were closely associated with positive attributes, indicating excellent initial sensory quality. Over time, sensory quality diverged among treatments. Samples PV2 and PPA at Day 45 retained desirable sensory characteristics, including a fresh aroma and a mild, characteristic flavor. However, at 90 days of storage, the PPA sample exhibited a strong shift toward highly negative attributes, such as unpleasant color and aroma, while PV3, at both 45 and 90 days, was closely associated with unpleasant taste. These findings are consistent with the lipid and protein oxidation data (TBARS and TVB‐N values), which indicated markedly higher oxidative degradation in PPA and PV3 samples, directly impacting their sensory profiles (Figures 2 and 3). Although PV2 and PV1 also experienced some sensory decline with storage time, the extent of deterioration was comparatively less pronounced. Notably, CATA results showed that shrimp reared under the same semi‐intensive system (PV1 and PV2) but in different regions developed distinctly different sensory profiles, highlighting the substantial influence of regional factors on sensory characteristics. In addition, the greater dispersion and less favorable positioning of wild‐caught samples (PPA) in the multivariate space reflect the inherent heterogeneity of this production system, which should be interpreted as a characteristic of the product rather than as experimental variability.

The multivariate interpretation was further supported by the Cochran's *Q* test, followed by multiple pairwise comparisons using the Marascuilo procedure (Calanche Morales et al. 2021), which identified attributes that significantly discriminated samples within each storage time (Table 3). Most attributes did not differ significantly among treatments (*p* > 0.05), indicating a similar overall sensory profile. However, specific attributes, particularly those related to color and flavor, showed significant differences and contributed to sample discrimination. Visual attributes, such as pinkish/orange and pale colors, were among the most discriminating parameters (*p* < 0.05) confirming differences in visual quality among production systems. In addition, flavor‐related attributes, including sweet, mild, characteristic, marine, and earthy notes, also differed significantly among treatments, reinforcing the role of production system and storage time in shaping sensory perception. The attribute “unpleasant” (aroma and flavor) showed significant differences (*p* < 0.05), particularly at intermediate storage times, indicating the onset of sensory deterioration. These results are consistent with the CA outcomes and corroborate the relationship between physicochemical changes and sensory perception. Furthermore, although differences became more evident over storage, the relatively limited number of significant attributes suggests that sensory changes were selective rather than generalized across all descriptors.

**TABLE 3:** Frequency of CATA sensory attributes for shrimp from different production systems during storage with Cochran's *Q* test and Marascuilo multiple comparisons.

Attributes	*p*‐Value	Day 1				Day 45				Day 90			
		PV1	PV2	PV3	PPA	PV1	PV2	PV3	PPA	PV1	PV2	PV3	PPA
Aroma													
Fresh	0.04	0.442 (a)	0.462 (a)	0.442 (a)	0.538 (a)	0.365 (a)	0.442 (a)	0.385 (a)	0.327 (a)	0.288 (a)	0.269 (a)	0.231 (a)	0.385 (a)
Ammonia‐like	0.626	0.038 (a)	0.038 (a)	0.038 (a)	0.019 (a)	0.077 (a)	0.077 (a)	0.077 (a)	0.058 (a)	0.038 (a)	0.019 (a)	0.038 (a)	0 (a)
Characteristic	0.485	0.519 (a)	0.462 (a)	0.538 (a)	0.519 (a)	0.327 (a)	0.442 (a)	0.365 (a)	0.385 (a)	0.462 (a)	0.404 (a)	0.442 (a)	0.423 (a)
Sweet	0.719	0.115 (a)	0.192 (a)	0.154 (a)	0.192 (a)	0.212 (a)	0.212 (a)	0.154 (a)	0.192 (a)	0.192 (a)	0.231 (a)	0.115 (a)	0.096 (a)
Mild	0.827	0.327 (a)	0.288 (a)	0.346 (a)	0.212 (a)	0.212 (a)	0.269 (a)	0.250 (a)	0.250 (a)	0.192 (a)	0.250 (a)	0.231 (a)	0.288 (a)
Unpleasant	0.016*	0.096 (ab)	0.096 (ab)	0.038 (b)	0.058 (ab)	0.154 (ab)	0.135 (ab)	0.135 (ab)	0.231 (a)	0.077 (ab)	0.077 (ab)	0.077 (ab)	0.019 (b)
Pleasant	0.453	0.212 (a)	0.404 (a)	0.346 (a)	0.346 (a)	0.327 (a)	0.327 (a)	0.308 (a)	0.192 (a)	0.365 (a)	0.269 (a)	0.288 (a)	0.288 (a)
**Color**													
Pinkish/Orange	0.000*	0.788 (a)	0.577 (abc)	0.750 (a)	0.519 (abc)	0.750 (a)	0.404 (bc)	0.769 (a)	0.346 (c)	0.769 (a)	0.500 (abc)	0.712 (ab)	0.404 (bc)
Pale	0.000*	0.135 (cde)	0.346 (bcde)	0.077 (de)	0.462 (ab)	0.192 (bcde)	0.500 (a)	0.135 (cde)	0.404 (abc)	0 (e)	0.288 (bcde)	0.135 (cde)	0.288 (bcde)
Bright	0.047*	0.288 (ab)	0.192 (ab)	0.269 (ab)	0.250 (ab)	0.231 (ab)	0.250 (ab)	0.308 (ab)	0.154 (ab)	0.346 (a)	0.154 (ab)	0.212 (ab)	0.077 (b)
Dull	0.012	0.115 (a)	0.135 (a)	0.077 (a)	0.231 (a)	0.212 (a)	0.269 (a)	0.135 (a)	0.212 (a)	0.038 (a)	0.154 (a)	0.058 (a)	0.192 (a)
Pleasant	0.013	0.481 (a)	0.423 (a)	0.519 (a)	0.308 (a)	0.308 (a)	0.250 (a)	0.346 (a)	0.269 (a)	0.538 (a)	0.346 (a)	0.404 (a)	0.327 (a)
**Flavor**													
Sweet	0.003*	0.269 (ab)	0.442 (a)	0.423 (a)	0.269 (ab)	0.173 (ab)	0.308 (ab)	0.231 (ab)	0.288 (ab)	0.327 (ab)	0.365 (ab)	0.250 (ab)	0.096 (b)
Salty	0.061	0.173 (a)	0.038 (a)	0.096 (a)	0.058 (a)	0.135 (a)	0.173 (a)	0.231 (a)	0.173 (a)	0.115 (a)	0.058 (a)	0.096 (a)	0.115 (a)
Mild	0.031*	0.442 (ab)	0.558 (a)	0.365 (ab)	0.481 (ab)	0.462 (ab)	0.385 (ab)	0.231 (b)	0.423 (ab)	0.327 (ab)	0.442 (ab)	0.288 (ab)	0.481 (ab)
Bitter	0.107	0.077 (a)	0.019 (a)	0.058 (a)	0.038 (a)	0.058 (a)	0.038 (a)	0.135 (a)	0.077 (a)	0 (a)	0 (a)	0.096 (a)	0.058 (a)
Unpleasant	0.014*	0.173 (ab)	0.058 (b)	0.154 (ab)	0.058 (b)	0.115 (ab)	0.096 (ab)	0.288 (a)	0.115 (ab)	0.154 (ab)	0.096 (ab)	0.212 (ab)	0.077 (ab)
Marine	0.003*	0.288 (ab)	0.231 (ab)	0.212 (ab)	0.288 (ab)	0.346 (ab)	0.173 (ab)	0.423 (a)	0.192 (ab)	0.250 (ab)	0.077 (b)	0.173 (ab)	0.173 (ab)
Characteristic	0.001*	0.385 (ab)	0.462 (a)	0.346 (ab)	0.404 (ab)	0.288 (ab)	0.308 (ab)	0.365 (ab)	0.231 (ab)	0.212 (ab)	0.192 (ab)	0.115 (b)	0.212 (ab)
Earthy	0.001*	0.173 (abc)	0 (c)	0.096 (abc)	0.058 (abc)	0.269 (a)	0.038 (bc)	0.250 (ab)	0.135 (abc)	0.115 (abc)	0.058 (abc)	0.154 (abc)	0.115 (abc)
Metallic	0.029	0.058 (a)	0 (a)	0.019 (a)	0.019 (a)	0.038 (a)	0.058 (a)	0.115 (a)	0.077 (a)	0 (a)	0 (a)	0.019 (a)	0.019 (a)
Pleasant	0.174	0.346 (a)	0.500 (a)	0.442 (a)	0.423 (a)	0.308 (a)	0.385 (a)	0.385 (a)	0.346 (a)	0.423 (a)	0.327 (a)	0.231 (a)	0.481 (a)
**Texture**													
Soft	0.382	0.423 (a)	0.481 (a)	0.500 (a)	0.481 (a)	0.462 (a)	0.404 (a)	0.442 (a)	0.481 (a)	0.346 (a)	0.481 (a)	0.558 (a)	0.615 (a)
Fibrous	0.0001*	0.192 (ab)	0.115 (b)	0.154 (ab)	0.212 (ab)	0.404 (a)	0.346 (ab)	0.269 (ab)	0.115 (b)	0.154 (ab)	0.212 (ab)	0.115 (b)	0.115 (b)
Rubbery	0.11	0.154 (a)	0.173 (a)	0.154 (a)	0.096 (a)	0.250 (a)	0.308 (a)	0.288 (a)	0.154 (a)	0.173 (a)	0.212 (a)	0.212 (a)	0.096 (a)
Juicy	0.22	0.327 (a)	0.404 (a)	0.385 (a)	0.308 (a)	0.250 (a)	0.288 (a)	0.173 (a)	0.212 (a)	0.269 (a)	0.385 (a)	0.308 (a)	0.269 (a)
Firm	0.171	0.231 (a)	0.385 (a)	0.288 (a)	0.404 (a)	0.288 (a)	0.327 (a)	0.346 (a)	0.269 (a)	0.308 (a)	0.192 (a)	0.173 (a)	0.231 (a)

*Different letters denote significant differences among treatments (*p* < 0.05).

To complement the descriptive insights from the CATA, a consumer acceptance test was performed. To better interpret consumer responses, a segmentation analysis was conducted, and the sociodemographic characteristics of the identified clusters are presented in Table 4. The distribution of consumers across clusters revealed a predominance of Cluster I, which accounted for the majority of participants, while Clusters II and III represented smaller consumer segments. Differences among clusters were mainly associated with age distributions, indicating distinct demographic profiles that influence consumer perception. In contrast, gender distribution was relatively balanced across clusters, suggesting that gender was not a determining factor in segmentation. The presence of distinct consumer groups highlights the heterogeneity of acceptance patterns, supporting the need for cluster‐based analysis rather than relying solely on overall mean scores. This segmentation approach provides a more realistic interpretation of consumer behavior and helps explain variations in acceptance observed across treatments and storage times.

**TABLE 4:** Sociodemographic characteristics of consumers by cluster and storage time (*n* = 100; data expressed as *n* (%), percentages calculated within clusters).

Storage times (days)	Sociodemographic variable	Category	Cluster I (73)	Cluster II (18)	Cluster III (9)
**1**	**Age**	18–25	48.5	11.3	68.0
		26–35	28.0	56.7	0
		36–45	5.6	11.3	16.0
		46–55	11.2	0	0
		> 55	6.7	20.7	16.0
	**Gender**	Female	65.3	68.0	67.5
		Male	34.7	32.0	32.5
**45**	**Age**	18–25	67.2	65.4	66.7
		26–35	13.4	21.8	0
		36–45	5.9	0	22.2
		46–55	8.1	0	0
		> 55	5.4	12.8	11.1
	**Gender**	Female	51.7	45.6	43.6
		Male	48.3	54.4	56.4
**90**	**Age**	18–25	60.3	66.7	44.4
		26–35	13.7	22.3	55.6
		36–45	13.7	11.0	0
		46–55	2.8	0	0
		> 55	9.5	0	0
	**Gender**	Female	79.5	55.6	44.4
		Male	20.5	44.4	55.6

*Note*: Frequency (%) is based on responses from 100 consumers.

Consumer acceptance results revealed distinct preference patterns among clusters (Table 5), highlighting the heterogeneity of consumer responses. Cluster I representing the majority of consumers, showed a gradual decline in acceptability over storage time, with scores decreasing from approximately 7.0–7.6 at Day 1 to 5.4–5.9 at Day 90. This trend is consistent with the physicochemical deterioration observed during storage, including increases in lipid and protein oxidation (Figures 2 and 3) and a shift toward negative sensory attributes identified by CATA (Figure 4).

**TABLE 5:** Overall acceptability of cooked shrimp during frozen storage (1, 45, and 90 days) by consumer clusters.

Treatments	Storage days	Cluster I (73)	Cluster II (18)	Cluster III (9)
**PV1**	1	7.6^a^	7.2^a^	7.8^a^
	45	6.9^ab^	6.2^abc^	7.0^a^
	90	5.4^c^	3.6^d^	6.6^a^
**PV2**	1	7.2^a^	7.1^a^	7.2^a^
	45	6.9^ab^	6.7^ab^	5.4^ab^
	90	5.9^bc^	3.9^cd^	5.0^ab^
**PV3**	1	7.0^ab^	7.2^a^	6.8^a^
	45	6.8^ab^	7.1^a^	5.2^ab^
	90	5.8^bc^	4.6^bc^	4.6^b^
**PPA**	1	7.0^ab^	6.2^abc^	6.0^ab^
	45	7.0^ab^	6.1^abc^	6.0^ab^
	90	5.8^bc^	5.7^abc^	5.0^ab^
**SIG**		***	***	***
**SEM**		0.2	0.5	0.8

*Note*: Shrimp were obtained from four production systems—semi‐intensive farming of *Penaeus vannamei* in Santa Catarina (PV1) and Ceará (PV2), organic farming of *P. vannamei* in Santa Catarina (PV3), and wild‐caught *P. paulensis* in Santa Catarina (PPA), Brazil. Sensory evaluation of cooked shrimp was conducted using a 9‐point hedonic scale (1 = *dislike extremely*, 9 = *like extremely*). Values are based on the responses of 100 consumers. Different letters in the same column indicate significant differences according to Tukey's test (*p* < 0.05).

Abbreviations: SEM, standard error of the mean; SIG, significance.

***p < 0.001.

In contrast, Cluster II exhibited a sharper decrease in acceptance, with scores dropping to 3.6–4.6 at Day 90, indicating greater sensitivity to quality deterioration. However, no significant differences among treatments were observed within this cluster. This behavior suggests that this group of consumers is more responsive to the emergence of negative sensory attributes during storage. Conversely, Cluster III maintained relatively high acceptance scores even at advanced storage times, indicating lower sensitivity to sensory changes and a greater tolerance to quality deterioration. This divergence across clusters demonstrates that consumer perceptions of product quality are not uniform and depend strongly on individual sensitivity.

Differences among treatments were strongly dependent on consumer cluster and were generally limited. In Cluster I, which represents the majority of consumers (73%), no significant differences among treatments were observed at any storage time, indicating that the production system did not influence acceptance for most consumers. A similar pattern was observed in Cluster II, where acceptability did not differ among treatments throughout storage. This reinforces that, for a substantial portion of consumers, product evaluation was not driven by differences among production systems. In Cluster III, no differences among treatments were detected at Days 1 and 45. However, at Day 90, PV3 showed lower acceptance than PV1. This indicates that treatment‐related differences became perceptible only under prolonged storage and within a small, more discriminating consumer segment.

Whereas most previous sensory studies on shrimp, primarily conducted on *P. vannamei*, have focused on the effects of diet formulation (Weldon et al. 2023; Zakarya et al. 2025) or processing interventions such as edible coatings (Mehraie et al. 2023), thermal treatments (C. Zhang et al. 2024), temperature fluctuation (S. Liu et al. 2024), preservatives (Yu et al. 2025; Zhu et al. 2024), and irradiation (Y. Zhao et al. 2023). The present study expands this approach by demonstrating that consumer acceptance is strongly modulated by segmentation and that differences among production systems are not uniformly perceived across consumer groups.

Lastly, shrimp (wild‐caught or farmed) incorporate local and regional environmental characteristics into their organoleptic properties (Ohto et al. 2021). As previously discussed (Sections 3.1 and 3.2), differences in environmental conditions, including temperature, feeding strategies, and pond characteristics, directly influence shrimp composition and quality (Gameiro et al. 2022; Bernardes et al. 2020). In addition, shrimp raised without supplemental feed, such as those from organic systems, rely exclusively on natural productivity, which is strongly influenced by soil composition and primary productivity (Lemonnier et al. 2017). These factors contribute to differences in chemical composition and sensory attributes among treatments.

The comparison between CATA (Figure 4) and consumer acceptance (Table 5) demonstrates the complementary nature of these approaches. Although CATA analysis revealed the progressive emergence of negative sensory attributes, these changes were not consistently reflected in consumer acceptance. This indicates that sensory modifications do not necessarily translate into rejection, as consumer response depends on the intensity of sensory changes and individual sensitivity. Therefore, physicochemical deterioration, sensory perception, and consumer acceptance are not linearly related but mediated by consumer segmentation and production conditions.

## 4 Conclusion

This study provided an integrated assessment of how production systems, semi‐intensive, organic, and wild‐caught affect the nutritional composition, lipid profile, oxidative stability, and sensory quality of *P. vannamei* and *P. paulensis* during 90 days of frozen storage. By combining proximate analysis, fatty acid profiling, lipid and protein degradation indices, and consumer‐centered sensory evaluation, the findings offer a robust basis for understanding system‐specific strengths and vulnerabilities.

Organic and wild‐caught shrimp delivered superior nutritional profiles, particularly in long‐chain omega‐3 fatty acids and favorable n‐6/n‐3 ratios, reinforcing their value as health‐promoting seafood. However, these same lipid traits increased susceptibility to oxidative and sensory deterioration during storage, indicating a need for targeted antioxidant strategies, whether dietary or through post‐harvest treatments, when aiming to preserve quality over time.

Semi‐intensive systems, particularly PV1, balanced nutritional quality with greater oxidative stability and visual appeal throughout storage, suggesting that feed formulation and controlled rearing environments can be optimized to achieve both health and technological performance goals. Differences between PV1 and PV2 further highlight the influence of region‐specific variables, feed composition, pond management, and environmental conditions on product quality.

From a practical standpoint, the results emphasize that production system choice should not be guided solely by nutritional output, but also by resilience to storage and processing conditions. Tailoring pre‐harvest strategies (e.g., feed lipid composition and inclusion of natural antioxidants) and post‐harvest handling to the oxidative risk profile of each system can enhance both market value and consumer acceptance, thereby supporting more sustainable and competitive shrimp aquaculture.

## Author Contributions

**Sarita Correa Rosa**: conceptualization, methodology, investigation, formal analysis, writing – original draft. **Milena Padilha**: methodology, investigation, validation, visualization. **Bibiana Alves dos Santos**: investigation, methodology, validation, visualization. **Pamela Cristiele Oliveira Trindade**: investigation, methodology, validation, visualization. **Géssica Hollweg**: investigation, methodology, validation, visualization. **Manoela Meira Balzan**: investigation, methodology, validation, visualization. **Priscila Rossato Fracari**: investigation, methodology, validation, visualization. **Alexandre José Cichoski**: investigation, methodology, validation, visualization, writing – review and editing. **Roger Wagner**: investigation, methodology, validation, visualization, writing – review and editing. **Natalia Fernandes Pereira**: investigation, methodology, validation, visualization. **Giovanni Lemos de Mello**: investigation, validation, visualization, conceptualization, methodology, funding acquisition, writing – review and editing. **Márcio Vargas‐ramella**: conceptualization, methodology, supervision, formal analysis, funding acquisition, writing – review and editing, project administration. **Paulo Cezar Bastianello Campagnol**: conceptualization, methodology, funding acquisition, writing – original draft, project administration, supervision.

## Conflicts of Interest

The authors declare no conflicts of interest.
